# The dynamics of HIV transmission in out of school young heterosexual men in South Africa: a systematic scoping review protocol

**DOI:** 10.1186/s13643-016-0398-y

**Published:** 2017-01-17

**Authors:** Nonzwakazi Ntombela, Tivani P. Mashamba-Thompson, Andile Mtshali, Anna Voce, Ayesha B. M. Kharsany

**Affiliations:** 1 Discipline of Public Health Medicine, School of Nursing and Public Health, University of KwaZulu-Natal, Durban, South Africa; 2 Centre for the AIDS Programme of Research in South Africa, Doris Duke Medical Research Institute, Nelson R Mandela School of Medicine, University of KwaZulu-Natal, Durban, South Africa

**Keywords:** Sex behavior, HIV transmission, Heterosexual men

## Abstract

**Background:**

In South Africa, gender inequality dominated by males and heterosexual HIV epidemic are associated with high HIV infection. Underlying epidemiological and social determinants driving HIV acquisition and transmission are critical to understand the extent and complexity of sexual networks as primary mechanisms through which HIV is likely to spread. The aim of the study is to provide an overview of empiric evidence that links the complex interaction of risk of HIV infection in men.

**Methods and analysis:**

We will conduct a systematic scoping review to identify, describe, and map literature on the dynamics of HIV infection in men, and we will determine the quality of the studies reporting on the dynamics of HIV infections in men. Primary research articles, published in peer-reviewed journals, review articles, and gray literature that address the research question, will be included. We will search PubMed, Web of Knowledge, Science Direct, EBSCOhost, Google Scholar, World Health Organization library, and UNAIDS database. Reference lists and existing networks such as government organizations and conferences will also be included to source relevant literature. Two independent reviewers will extract data in parallel from all relevant search engines, using specific inclusion and exclusion criteria. A thematic content analysis will be used to present the narrative account of the reviews, using NVivo version 10.

**Discussion:**

We anticipate finding relevant literature on the dynamics of HIV transmission in South African men. Once summarized, data will be useful to guide future research.

**Systematic review registration:**

PROSPERO CRD42016039489

**Electronic supplementary material:**

The online version of this article (doi:10.1186/s13643-016-0398-y) contains supplementary material, which is available to authorized users.

## Introduction

South Africa accounts for 18% of global HIV infections, with approximately 6.7 million people infected [1]. Each day, there are thousands of new HIV infections [2]. With only limited bio-behavioral intervention focusing on men [3, 4], South Africa may be far from altering the epidemic trajectory. Strategies to reduce HIV transmission in men would benefit greatly from a better understanding of the sexual networks that drive HIV transmission.

For decades, HIV surveillance and preventive intervention research has largely focused primarily on females [5–11]. Although this population has received overwhelming attention, the prevalence and the rates of new infections continue to increase. With high gender inequalities in South Africa, being one explanation, it has made it difficult for females to take control in their sexual encounters. In the South African context, especially in Black Africans, gender norms play a crucial role in sexual relationships with males taking precedence in decision-making, and views from men are respected by most women [12]. While men are perceived to be “heads,” they tend to engage in multiple risks, predisposing their sexual partners to HIV infection, and with their views, may influence the success or failure of any HIV prevention programs for both males and females. Males therefore may play a critical role when developing HIV reduction interventions.

Despite the high coverage of HIV counseling and testing (HCT) and antiretroviral therapy services in South Africa, a high number of people remain uninformed about their HIV status, majority being males, and do not perceive themselves as being susceptible to HIV infections and may therefore not take preventive measures to reduce HIV transmission and faster progression of acquiring infection to AIDS defining illness [13]. Published data suggests increasing HIV risk behaviors in men [3] and suggests that being male, or having low level of education, or being Black African, or being from a rural area, or being unemployed, are associated with low knowledge of own HIV status [4]. Having more than four lifetime partners, intercourse under the influence of alcohol or drug use, transactional sex [14], and being between 15–35 years [6, 14, 15] are associated with HIV infection in men. Moreover, the interaction of early sexual debut, low condom use, multiple sex partners either concurrently or sequentially, and age disparate relationships enhance the transmissibility and propagation of HIV in heterosexual relationships [13, 16]. However, lack of in-depth data on the contribution and the interaction of the drivers of the epidemic may not be well measured and therefore fail to accurately determine association and temporality.

The presented literature suggests the importance of understanding of HIV transmission dynamics in males and underscores the need for focused research to address this major gap in the knowledge of HIV epidemiology. The interactions of structural, behavioral, and biological risk factors of HIV acquisition and/or transmission are poorly understood and are a key to the design of HIV intervention programs. However, it is imperative that we understand the structural features of risks of HIV transmission in sexual networks as very little work has been done on characterizing these structures. Large complex studies are required to understand these risks, and as there exists no data for a clearer understanding on these complex interactions of risks. The purpose of the scoping review is to refine a future research agenda and provide directions to future research by establishing to what extent existing research has progressed towards understanding the dynamics of HIV in South Africa.

The primary aim of the study is to map existing literature on the dynamics of HIV transmission in men in South Africa. In order for us to achieve the study aim, the following objectives are set:

1. To identify and describe the epidemiology of HIV in out of school men
2. To identify and describe HIV transmission dynamics in out of school men
3. To determine the nature and quality of studies reporting evidence on dynamics of HIV in men

## Materials and methods

The current scoping review protocol has been prepared in consultation with the PRISMA-P statement [17] (see Additional file 1). It has been registered and published in the PROSPERO international prospective register for systematic reviews. It is registered under the following registration number: CRD42016039489.

### Scoping review framework

The framework adopted for conducting the proposed review is by Arksey, H., and O’Malley, [18]. Briefly, the framework involves (I) identifying the research question, (II) identifying relevant studies, (III) study selection and (IV) charting the data, and (V) collating, summarizing, and reporting the results.

I. Identifying the research question

The research question is: What is known from existing literature about the dynamics of HIV transmission in men in South Africa?

The research sub-questions are:

1. What is the prevalence and incidence of HIV infection in out of school men of South Africa?
2. Is there a relationship between the social networks and sexual networks for HIV-1 transmission in out of school men of South Africa?
3. What interactions exist between biological, behavioral, and cultural factors in HIV-1 sexual networks?

#### Eligibility of research question

The study will use an amended PICOS (population, intervention, comparison, outcomes and study setting) framework to determine the eligibility of the research question, as described in Additional file 2: Table S1. Additional file 2: Table S1 is provided in the supplemental documents to this protocol, as Additional file 2.

II. Identifying relevant studies

Primary research articles, published in peer-reviewed journals; review articles; and gray literature that address the research question, will be included in this study. All study designs will be included for review. Databases that will be used to source literature include; PubMed, Web of Knowledge, Science Direct, EBSCOhost, Google Scholar, World Health Organization library database, and UNAIDS database. We will also use reference lists and existing networks such as organizations and conferences to source relevant literature. The search terms will include “HIV infection in men and sex behaviors, or cultural factors, or social networks, or sexual networks, or phylogenetic or biological factors”. The language and year of publication will be restricted to English and between 2006–2016, respectively.

III. Study selection

One reviewer will conduct a comprehensive title screening by searching and uploading all literature search results on Endnote X7 software; all studies that do not address our research question will be excluded together with all duplicates. The final Endnote database will be shared for abstract screening; at this stage, we will use two independent reviewers to extract data in parallel, from all relevant search engines. The two reviewers will use the inclusion/exclusion criteria to identify relevant literature for inclusion and for further evaluation (s). Copies of full articles will be obtained and maintained.

Inclusion/exclusion criteria, developed based on the research question will ensure correct identification and selection of relevant studies. To be included, studies must be focusing on HIV infection, transmission, or acquisition on heterosexual males. We will include both peer-reviewed publications and gray literature of all study designs with relevant interventions. We will exclude studies on HIV infections due to mother to child transmission of HIV and studies on high-risk men (e.g., men who have sex with men, gays, bisexuals, injection drug users, male sex workers, and transgender populations). The search strategies will be piloted to test the appropriateness of the selected databases and keywords in delivering relevant information/literature. The study selection procedure will be summarized using a PRISMA chart (Additional file 3: Figure S1), modified from [19].

IV. Charting the data

We will use a standardized data extraction sheet. The sheet will include bibliographic details, study design, number of participants, intervention (s), study setting, and conclusions for the primary and the secondary outcomes of the interventions. An additional charting summary Additional file 4: Table S2 shows this in more detail [see Additional file 4].

V. Collating, summarizing, and reporting the results

We will present a narrative account of findings from existing literature through thematic content analysis of the extracted literature, structured around the following interned outcomes: HIV transmission, HIV acquisition, and HIV infection. Results of the studies on HIV infection, acquisition, or transmission in heterosexual men outside South Africa vs South African men, on evidence of whether interaction of interventions leads to HIV infection (transmission or acquisition), will be coded by all authors independently.

We use NVivo version 10 to present emerging themes according to the relevant interventions.

The below processes will be followed:

- Coding
- Categorize codes into major themes
- Build theme-related themes (cut-and-paste technique)
- Display data
- Identify patterns in the data and identify sub-themes
- Summarize

Authors will interrogate the resulting themes and critically examine their relationship to the research question. Authors will also scrutinize the meanings of the findings as they relate to the overall aim of the study and address the implications for future research.

### Quality assessment

Mixed methods tool will be used to assess the quality of evidence obtained from the search. The mixed method quality appraisal tool obtained from Mixed Methods Appraisal Tool (MMAT) Version 2011 [20] will be utilized.

## Discussion

The current scoping review will provide evidence of the existence of complex interactions of risks undertaken by men, leading them to high risks to HIV acquisition and transmission and will provide evidence to refine future research agenda and provide directions to future research. While evidence suggest being a young male, having low level of education, unemployed, being Black African, and from a rural area predisposes males to high-risk behaviors for HIV transmission [4, 14, 16], little is known on the complex interaction of risks in sexual networks. With the UNAIDS aiming to reach the 90-90-90 goals by 2020 [21], such information is highly valuable. If we aim to have 90% of people living with HIV know their status, 90% diagnosed receiving ART, and 90% of those achieve viral suppression, we need to understand the epidemiological and social interactions of risks that may prevail South Africa from achieving the 90-90-90s.

With South Africa being predominated by unequal gender norms dominated by men, although importantly focusing on females, a holistic approach is needed to end the trajectory. It is imperative that we incorporate males in research to better understand the issues revolving around them and perhaps leading to their increased risk-taking behaviors. We anticipate finding relevant literature on the dynamics of HIV transmission in South African men. We will extend our review to include other countries to compare findings from other countries to those of South Africa. We will also include studies that focus on both males and females, but we will target results reporting on males. Because a huge amount of HIV research is produced globally each year, and we are interested on current and potentially relevant studies, we will limit our search to include the latest published studies from 2006–2016. It is essential to have a comprehensive and balanced account of previous work to provide a sound background of the research. It is because of this reason that the study will make use of a 10-year literature search. It will clearly define perspectives that have emerged and changed through the years as there has been extensive HIV research and interventions that have been conducted in the past 10 years. The purpose is to familiarize the reader with existing studies relevant to the gap in the knowledge, and a time shorter than the specified might not be sufficient to determine sustainable behavior in the targeted audience.

The results will add an overview of documented evidence on complex interaction of risks in men and the dynamics of HIV transmission and will help identify requirement priorities for primary research in this area.

## Additional files

Additional file 1: PRISMA-P checklist. (DOCX 29 kb)
Additional file 2: Table S1. Framework for determining eligibility of research questions. (DOCX 13 kb)
Additional file 3: Figure S1. PRISMA chart of data extraction. PRISMA chart allows for transparent reporting of systematic reviews and meta-analysis. Data will be extracted at various stages of the chart through screening titles, abstracts, and full text. Articles failing to meet the inclusion criteria will be excluded at any stage of screening. (PDF 272 kb)
Additional file 4: Table S2. Charting summary table. (DOCX 11 kb)

## Abbreviations

AIDS: Acquired immunodeficiency syndrome
ART: Antiretroviral therapy
HCT: HIV counseling and testing
HIV: Human immunodeficiency virus
