# Supplementary material for: The dynamics of HIV transmission in out of school young heterosexual men in South Africa: a systematic scoping review protocol
Source: Syst Rev. 2017 Jan 17;6:9. doi: 10.1186/s13643-016-0398-y (PMC5240355; doi:10.1186/s13643-016-0398-y)
Supplement: Additional file 2: Table S1. — Framework for determining eligibility of research questions. (DOCX 13 kb) [file 13643_2016_398_MOESM2_ESM.docx]

| **Population:** | **Intervention:** | **Comparison** | **Outcomes:** | **Study setting:** |
| --- | --- | --- | --- | --- |
| Heterosexual men, and not attending school. | - Biological factors - Behavioral factors - Cultural factors - Social networks and - Sexual networks | Heterosexual men from outside South Africa | HIV transmission/ acquisition/ infection | Global |

Table S1: Framework for determining eligibility of research questions

PICOS framework will be used to determine the eligibility of the research question. The population of interest is heterosexual men not attending school. The study will examine the impact of biological, behavioral, cultural factors, as well as social and sexual networks on HIV transmission/ acquisition and infection. South African heterosexual men will be compared to heterosexual men from outside South Africa.
