# Supplementary figures and images for: The dynamics of HIV transmission in out of school young heterosexual men in South Africa: a systematic scoping review protocol
Source: Syst Rev. 2017 Jan 17;6:9. doi: 10.1186/s13643-016-0398-y (PMC5240355; doi:10.1186/s13643-016-0398-y)

Identification

Screening

Eligibility

Included

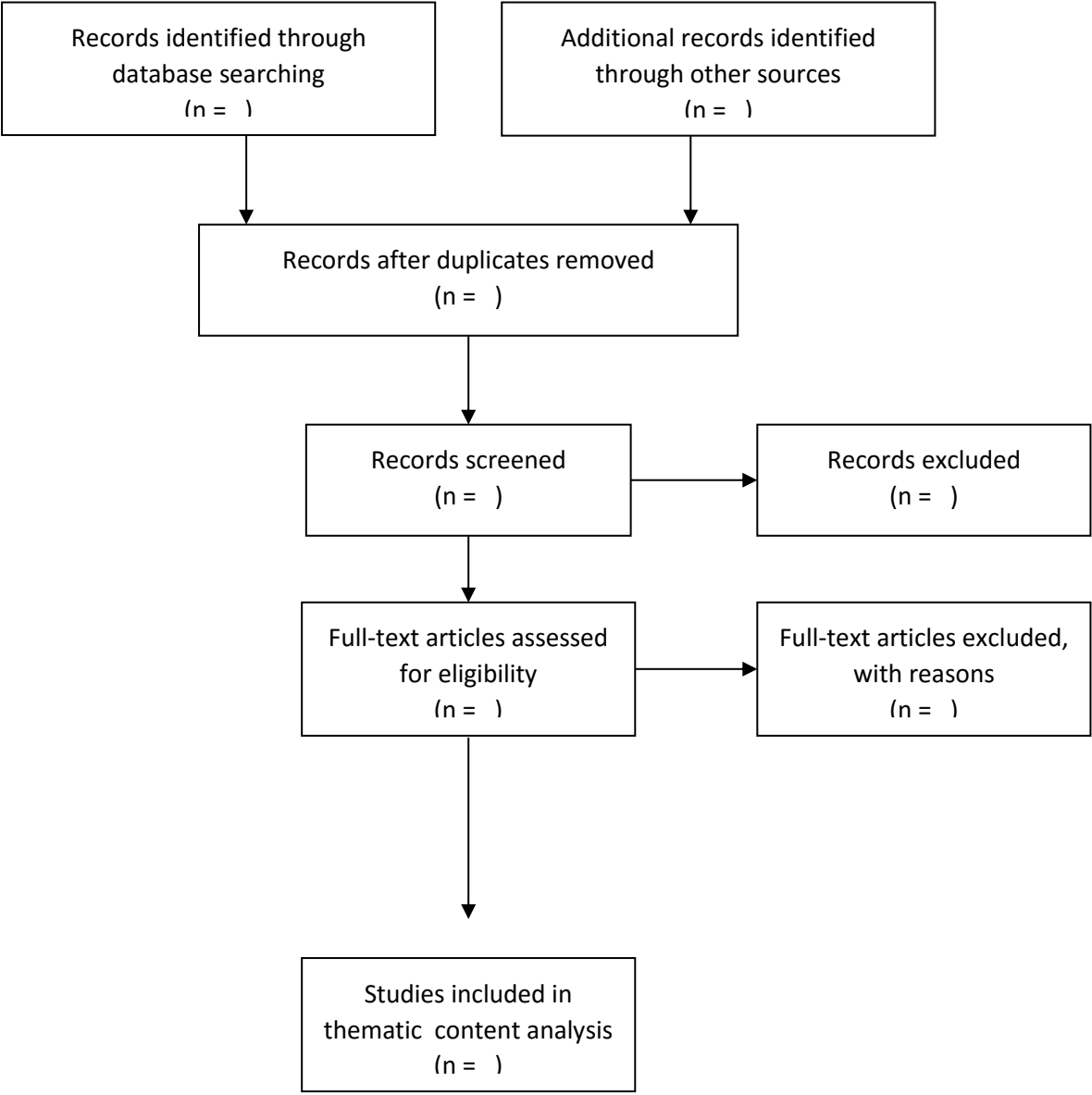

Supplement: Additional file 3: Figure S1. — PRISMA chart of data extraction. PRISMA chart allows for transparent reporting of systematic reviews and meta-analysis. Data will be extracted at various stages of the chart through screening titles, abstracts, and full text. Articles failing to meet the inclusion criteria will be excluded at any stage of screening. (PDF 272 kb) [file 13643_2016_398_MOESM3_ESM.pdf]
