# Supplementary material for: The dynamics of HIV transmission in out of school young heterosexual men in South Africa: a systematic scoping review protocol
Source: Syst Rev. 2017 Jan 17;6:9. doi: 10.1186/s13643-016-0398-y (PMC5240355; doi:10.1186/s13643-016-0398-y)
Supplement: Additional file 4: Table S2. — Charting summary table. (DOCX 11 kb) [file 13643_2016_398_MOESM4_ESM.docx]

| **Author and year** | **Study setting** | **Population** | **Intervention** | **% Heterosexual men** | **Age** | **Aims** | **Study design** | **Sample size** | **Outcome measure** | **Key finding** | **Comments** |
| --- | --- | --- | --- | --- | --- | --- | --- | --- | --- | --- | --- |
|  |  |  |  |  |  |  |  |  |  |  |  |

Table S2: Charting summary table

Data from all eligible studies will be extracted using a standardized charting summary table. Bibliographic details, study design, number of participants, intervention(s), study setting, and conclusions for the primary and secondary outcomes of interest will be extracted and displayed in the table.
